# The correlation between the bronchial hyperresponsiveness to methacholine and asthma like symptoms by GINA questionnaires for the diagnosis of asthma

**DOI:** 10.1186/1471-2466-14-161

**Published:** 2014-10-18

**Authors:** So Yeon Lim, Young Joo Jo, Eun Mi Chun

**Affiliations:** Division of Pulmonary and Critical Care Medicine, Department of Internal Medicine, Ewha Womans University School of Medicine, 1071 Anyangcheon ro Yangcheon-gu, Seoul, 158-710 Korea; Division of Allergy and Immunology, Department of Internal Medicine, Ewha Womans University School of Medicine, Seoul, Korea

**Keywords:** Questionnaire, Bronchial hyper responsiveness, Asthma like symptoms

## Abstract

**Background:**

In epidemiological studies of asthma, questionnaires to differentiate asthmatics from non-asthmatics have proven to be cost-effective and convenient. The aim of this study was to analyze the association between hyperresponsiveness to methacholine and the validity of five items for the asthma like questionnaire recommended by the Global Initiative for Asthma (GINA).

**Methods:**

A total of 680 subjects who visited the pulmonology department with suspected symptoms of asthma were enrolled. All participants completed five items questionnaires and underwent methacholine bronchial provocation tests (MBPT). The diagnostic value of the questionnaire was assessed through analysis of the sensitivity, specificity, and positive and negative predictive values.

**Results:**

Multivariate logistic regression analysis showed that questionnaires about wheezing, exercise induced dyspnea and pollution-induced dyspnea were useful for differentiating asthmatics from non-asthmatics (adjusted odds ratio (OR) =2.0, 95% confidence interval (CI) 1.3-3.0; OR =2.3, 95% CI 1.5-3.5; OR =2.0, 95% CI 1.3-3.0) respectively. A total symptom score of higher than 1 was associated with the highest sensitivity (98.4%) and lowest specificity (9.4%). In contrast, a total symptom score of more than 5 was associated with the highest specificity (91.9%) and lowest sensitivity (18.5%)

**Conclusions:**

Although questionnaires are not a sufficiently accurate method for diagnosing asthma, properly selected questionnaire can be used as effective strategies in situations such as private clinics or large population based epidemiologic studies.

## Background

The prevalence of asthma has increased continuously worldwide in recent decades [1]. Asthma is clinically diagnosed by physicians with asthma like symptoms of patients in community settings. However, asthma incidence cannot be determined precisely because there is no generally accepted gold standard definition of asthma. The Medical Research Council (MRC) developed a questionnaire to detect chronic bronchitis; a similar questionnaire was designed for asthma several years later [2]. Thereafter, large population-based epidemiological studies of asthma have usually relied on several types of questionnaires of symptom-based components, such as wheezing or tightness of the chest [3]. To increase the accuracy of epidemiological surveys of asthma, objective measurements of airway hyperresponsiveness have been used as supplements for diagnosing asthma [4]. International guidelines recommend that asthma should be suspected in patients with respiratory symptoms such as chronic cough, wheezing episodes, dyspnea, chest tightness and a positive bronchial hyperresponsiveness (BHR) [5]. Until recently, epidemiologic studies have generally relied upon the use of symptom-based questionnaires to distinguish asthmatics from non-asthmatics due to their convenience and cost-effectiveness [6, 7]. Therefore, most studies of the prevalence of asthma have used patient questionnaires inquiring about episodes of wheezing, dyspnea, and persistent cough [8]. However, this approach often fails to detect asthma accurately because most studies inquire about subjective symptoms; e.g., physicians and patients may interpret the term “wheeze” differently. Questionnaires alone can misjudge the prevalence of asthma due to the lack of a standard definition. Thus, epidemiological surveys that collect data using questionnaires often overestimate asthma prevalence [9]. In contrast, many patients with true asthma are diagnosed as non-asthmatics or are misdiagnosed with other respiratory illnesses. The most common characteristic of asthma is the hyperresponsiveness of the airway to the stimuli which generally cannot influence non-asthmatics. Previous studies have demonstrated that asthmatics are more likely to have BHR than non-asthmatics. In contrary, some studies reported that the presence of BHR cannot accurately discriminate asthmatics from non-asthmatics in population based studies [10]. Although BHR is not considered essential factor to diagnosis asthma due to low sensitivity, it is most available method to assess the validity of asthma diagnosed by questionnaires. Therefore, BHR is widely recognized as the standard diagnostic parameter for asthma in spite of clinical inaccuracy. Asthma might be diagnosed when there are both positive asthma symptoms and BHR [11]. The methacholine provocation test (MBPT) has been used universally to assess BHR in patients with asthma. The MBPT can be repeated easily and correlates relatively well with the presence and clinical severity of asthma [12]. Although MBPT is regarded as a standard method to confirm the presence of BHR, it has limitations precluding its use as the definitive tool for diagnosis of asthma. Although there is a predictable relationship between a positive BHR and asthma, BHR is not a highly sensitive or specific strategy for the clinical diagnosis of asthma [13]. Unfortunately, a negative response to the methacholine test does not completely exclude asthma. In addition, MBPT is also costly and time consuming to perform in epidemiological studies or in private clinics. To enhance the accuracy of questionnaires, scoring systems to identify asthma in large population surveys using a combination of predictor variables collected by questionnaires have been developed [14, 15]. Therefore, the present study was designed to validate the accuracy of five questions representing asthma like symptoms along with the MBPT, and to evaluate the clinical usefulness of this method in private clinics or large-population-based epidemiological surveys.

## Methods

### Participants and study design

Six hundred and eighty subjects were recruited from patients visited to the outpatient department with varied respiratory symptoms suggesting asthma, such as dyspnea, chronic cough, chest tightness and wheezing. Participants were mixed populations referred from other primary physicians and visited to pulmonary department by themselves without consultations. At the first visit, all subjects were asked to complete five asthma screening questionnaires developed based on common questions recommended by GINA guidelines regarding respiratory symptoms associated with asthma [16]. The answers to each question were recorded simultaneously and all questions could be answered with “yes” or “no”. The total symptom score was calculated by summing the scores corresponding to each question. Participants were divided into two groups of asthmatics and non-asthmatics. Participants were classified as asthmatics if the subjects were matched to the following criteria: 20% decrease in forced expiratory volume in 1 second (FEV_1_) with a dose of <16 mg/mL inhaled methacholine. Participants with negative results on the methacholine challenge test were regarded as non-asthmatics. Exclusion criteria were as follows: 1) current diagnosis of pneumonia, emphysema, tuberculosis or other lower respiratory tract diseases, and infections of the ear, sinus, or upper respiratory tract diseases, 2) uncontrolled cardiovascular diseases, malignancy, immunosuppressive diseases, 3) patients hospitalized within 3 months due to other respiratory diseases; 4) pregnant and breastfeeding women, and patients under 18 years old. The subjects having other lung diseases including pneumonia, emphysema, tuberculosis, interstitial lung disease were exclude by radiologic examinations.

### Procedures

Subjects who met the eligibility criteria for this study received informations about the protocols. Each participant who met the criteria answered the five questions, receiving help from nurses or physicians. All participants underwent basal spirometry (Sensor Medics, Yorba Linda, CA. USA). The following parameters were measured: FEV_1_, FVC, and FEV_1_/FVC. The highest FEV_1_ was selected among three consecutive procedures with basal spirometry. Subjects with a basal FEV_1_ of more than 70% of the predictive value by spirometry underwent MBPTs. Prior to the MBPT, subjects were asked to discontinue any medications that could interfere with the methacholine test. The diagnosis of asthma was confirmed based on a positive response to the MBPT (PC20 ≤ 16 mg/dL of inhaled methacholine). The incremental concentrations of methacholine chloride prepared from the dosing protocol were 0.0625, 0.25, 1, 4, 16, 25, and 50 mg/mL. A decrease of ≥20% of the baseline FEV_1_ with a dose of <16 mg/mL of methacholine was considered a positive response. Methacholine was inhaled using the 2-min tidal breathing method with a synchronized nebulizer or five-breath dosimeter method (DSM-2) according to ATS guidelines. Spirometry was repeated 3 min after each increased dose of methacholine. After the methacholine test, all participants received salbutamol and repeated spirometry was performed to assess recovery of lung function. Patients were divided into two groups, asthmatics and non-asthmatics, according to the results of the MBPT. Patients were diagnosed with asthma if their answers to the questionnaire suggested it and the MBPT was positive. The relationship between asthma symptoms and the presence of BHR was determined by the sensitivity (proportion of patients with BHR who had a positive questionnaire result) and specificity (proportion of patients with normal responsiveness who had a negative questionnaire result). The baseline characteristics of the asthmatics and non-asthmatics are shown in Table 1. This study protocol was approved by the Institutional Review Board (Approval No. ECT198-2-16) of Ewha Womans University Mokdong Hospital and we received written informed consent from participants.

**Table 1:** **Baseline characteristics of subjects who underwent MBPT and completed questionnaire**

Characteristic	Asthmatics	Non-asthmatics
	(n = 164)	(n = 516)
Mean age, years	43 (20–64)	49 (20–81)
Gender (male: female)	2:3	2:3
Body mass index, kg/m^2†^	23.5 ± 2.4 (17–30)	22.6 ± 2.4 (17–30)
Smoking history, number (%)		
Never smoked	96 (58)	296 (57)
Current smoker	22 (13)	120 (23)
Ex-smoker	2 (1)	42 (8)
FEV_1_ (%predicted)	93 (70–135)	98 (70–148)
FEV_1_/FVC (%predicted)	78 (70–95)	82 (70–99)

^†^P <0.05; compared with non-asthmatic patients by MBPT.

*Abbreviations*: *MBPT* methacholine bronchial provocation test, *FEV*_*1*_ forced expiratory volume in 1 second, *FEV*_*1*_ */ FVC* forced expiratory volume in 1 second/forced vital capacity.

### Asthma screening five-item questionnaire based on GINA

- Q1. Has the patient had an attack of wheezing?
- Q2. Does the patient have wheeze or dyspnea after exercise?
- Q3. Does the patient have a troublesome cough at night?
- Q4. Did the patient’s cold take more than 10 days to clear up?
- Q5. Did the patient experience wheezing, chest tightness, or cough after exposure to airborne allergens or pollutants?

### Statistical analysis

The mean total symptom scores for the two groups were compared using Student’s *t*-test. Multivariate logistic regression analysis was performed to determine whether the five questions used as independent variables could significantly differentiate asthmatics and non-asthmatics. The correlation between the questionnaire and asthma was defined by the odds ratios (OR) and 95% confidence intervals (CI). A receiver-operating characteristic (ROC) curve analysis was performed to assess the diagnostic accuracy of the symptom-assisted diagnosis. A p value less than 0.05 was considered to indicate statistical significance. Statistical analyses were performed using SPSS version 16.0 (SPSS, INC, Chicago, IL, USA).

## Results

**Figure 1:**
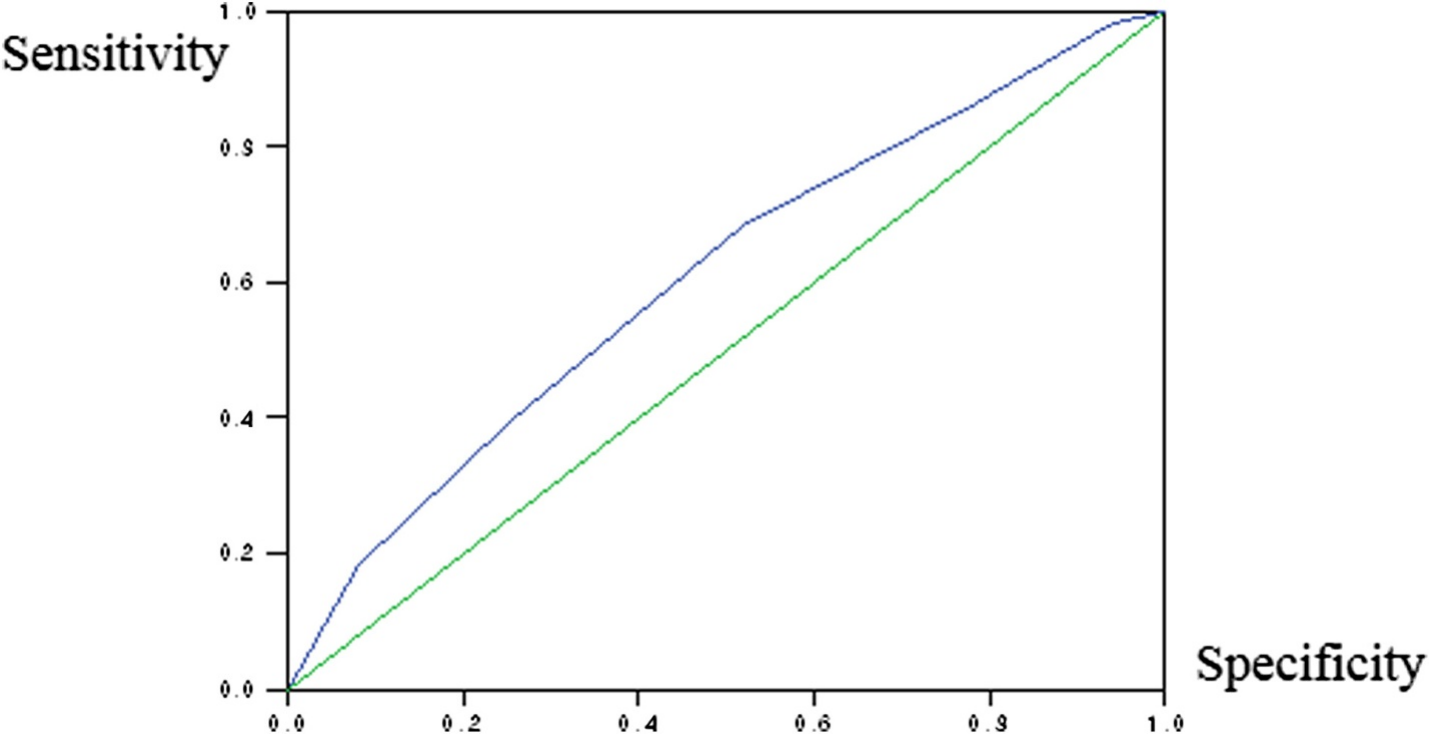
**Area under the receive operating curve (ROC) for the symptom score.** The AUC of the ROC curve was 0.610 ± 0.029. The probability of higher symptom scores for asthma group was 61% greater than for the control group.

Of the 680 subjects, 24% (n = 164) had asthma and 76% (n = 516) did not. Differences in the baseline clinical characteristics of asthmatics and non-asthmatics were not statistically significant, with the exception of the body mass index (BMI) (Table 1). The BMI of the asthmatics was higher than that of the non-asthmatics (mean 23.5 ± 2.4 vs. 22.6 ± 2.4, p <0.05). Table 2 shows the prevalence and predictive value of each question for diagnosing asthma. The exercise-induced dyspnea question had the highest sensitivity (70.2%) but a relatively low specificity (49.1%). By contrast, attacks of wheezing had the highest specificity (65.8%), but moderate sensitivity (50.8%). Five questionnaires showed high negative predictive values (NPV) of over 82% but low positive predictive values (PPV) of less than 28%. Table 3 shows the multivariate logistic regression analysis of the association between the questionnaire and the results of the MBPT. Exercise-induced dyspnea was the most significant questionnaire item that differentiated asthma patients from non-asthmatic patients (OR = 2.3, CI: 1.5 to 3.5, p <0.001). Recurrent attacks of wheezing and allergen or pollution induced dyspnea were also highly correlated with the diagnosis of asthma after adjusting for all symptoms (OR = 2.0, CI: 1.3 to 3.0, p <0.001). With an increase of the cutoff value from 1 to 5, the sensitivity decreased progressively (from 98.4% to 18.5%), while the specificity increased continuously (from 9.4% to 91.9%). A total symptom score of ≥3 was associated with moderate sensitivity (68.5%) and specificity (48%) (Table 4). Table 5 shows that a PC20 ≤ 50 mg/ml (62.4%) exhibited a slightly higher sensitivity than did a PC20 ≤ 25 mg/ml (44.2%); however, the predictability of PPV was similar for both methacholine doses. The diagnostic value of the questionnaire was evaluated by ROC analysis. The AUC of the ROC curve was 0.610 ± 0.029 (Figure 1). An AUC OF 0.6 appears that BHR in this cohort means modestly predictive of an increased symptom score for the asthma group.

**Table 2:** **Prevalence and predictive values of questions for diagnosing asthma by GINA**

Question	Prevalence (%)	Sensitivity (%)	Specificity (%)	PPV* (%)	NPV ^†^(%)
Q1. Wheezing	38	50.8	65.8	28.1	83.6
Q2. Exercise-induced dyspnea	53	70.2	49.1	26.7	86.2
Q3. Nocturnal cough/dyspnea	47	62.1	44.8	22.8	81.8
Q4. URI^‡^ ≥10 days	49	64.5	42.2	22.7	81.8
Q5. Pollution-induced dyspnea	50	66.1	39.7	22.4	81.7

*Abbreviations*: * *PPV* positive predictive value, ^†^ *NPV* negative predictive value. ^‡^ *URI* upper respiratory tract infection.

**Table 3:** **Multivariate logistic regression analysis of questions by GINA**

Question	Positive response		OR*	95% CI ^†^	P-value
	Asthma G	Control G			
Q1. Wheezing	63	161	2.0	(1.3-3.0)	<0.001
Q2. Exercise-induced dyspnea	87	239	2.3	(1.5-3.5)	<0.001
Q3. Nocturnal cough or dyspnea	77	260	1.3	(0.9-2.0)	0.169
Q4. URI ≥10 days	80	273	1.3	(0.9-2.0)	0.187
Q5. Pollution-induced dyspnea	63	161	2.0	(1.3-3.0)	<0.001

*Abbreviations*: *OR odds ratio, ^†^ *CI*, confidence interval.

**Table 4:** **Sensitivity and specificity of combined scores of each symptom for diagnosis of asthma by GINA**

Cutoff value	Sensitivity (%)	Specificity (%)
≥1	98.4	9.4
≥2	86.3	20.4
≥3	68.5	48.0
≥4	39.5	74.6
≥5	18.5	91.9

**Table 5:** **Prediction of asthma using PC20 values of ≤25 mg/ml and ≤50 mg/ml**

Sensitivity	Specificity	PPV*	NPV ^†^
44.2	75.2	88.5	24.0
62.4	52.2	84.8	24.7

*Abbreviations*: * *PPV* positive predictive value, ^†^ *NPV* negative predictive value.

## Discussions

The acceptable method to identify asthma patients seems to be a combination of asthma like symptoms and bronchial challenge test, in addition to a clinical diagnosis by a physician [17]. BHR is considered as a relatively standard diagnostic method for asthma but has several limitations. First, many subjects with BHR were asymptomatic; BHR has high sensitivity but low specificity as a diagnostic tool for asthma. MBPT frequently underestimates the sensitivity of the asthma questionnaire [18]. Second, MBPT is a costly and time-consuming method for use in a large population-based epidemiology survey. Therefore, the conventional questionnaire for detecting asthma has been used widely in epidemiological surveys due to its cost-effectiveness and convenience. However, there has not been developed a generally accepted questionnaire for diagnosing asthma until now. We attempted to overcome this limitation using a questionnaire that was properly correlated with the clinical symptoms of asthma. Although there have been a few reports concerning the validity of the respiratory questionnaire for detection of asthma, this paper is the first to validate the asthma questionnaire recommended by GINA in combination with the MBPT results of adult respiratory patients in Korea. Although obesity has been known to evoke or aggravate asthma in the general population, deteriorating airway hyper-responsiveness is not thought to do so [19–22]. In our study, the baseline characteristics of both groups were not statistically significantly different, with the exception of body mass index (BMI). Present study also demonstrated that obesity certainly play a role to increase the incidence of asthma. To validate the questionnaire, one must calculate the sensitivity and specificity. Sensitivity is the proportion of subjects truly diseased based on the questionnaire; specificity is the proportion of subjects to be healthy based on the questionnaire. Kilpelainen et al. reported the validation of a questionnaire for respiratory symptoms in patients with “current asthma”; wheezing with episodes of shortness of breath showed a high specificity (93%) and a moderate -to-low sensitivity (45%) [23]. In our study, wheezing showed a sensitivity of 50.8% and a specificity of 65.8%. Exercise-induced dyspnea had a sensitivity of 70.2% and a specificity of 49.1%. The possible causes of the comparatively low sensitivity of wheezing in our study are that wheezing is interpreted subjectively by patients and wheezing in asthma patients is sometimes underestimated due to confusion with other diseases producing wheezing, such as COPD and localized obstructive bronchial diseases (e.g. cancer, endobronchial lesions, etc.). Additionally, the underestimated effect of MBPT when combined with a questionnaire is a probably negative factor for the sensitivity of asthma. The other possible reason for the low sensitivity of wheezing is that it is determined usually by physicians rather than patients, and many asthma patients report no asthma symptoms despite a positive BHR. Jenkins et al. reported that questionnaires are valid instruments for the determination of asthma symptoms in the previous 12 months [24]. They reported that self -reported symptoms had a higher Youden’s Index than did BHR because of the greater sensitivity of questionnaires. Youden index, (*J*) = maximum {sensitivity( *c* ) + specificity( *c* )-1}, is generally used as the method of overall diagnostic effectiveness. The value close to 1 indicate that the biomarker’s effectiveness is relatively large [25]. Symptoms combined with the BHR showed increased specificity with a severely decreased sensitivity due to inclusion of the BHR. In general, many other population-based studies showed similar results to those of Jenkins et al. with a specificity of more than 90%, a sensitivity of 20-50%, and a Youden’s Index of less than ~40% [26–28]. They suggested that physician diagnosis of asthma used by questionnaire appears reasonable tool because diagnosis of BHR plus questionnaire usually decrease the incidence of asthma due to low sensitivity of BHR. The purpose of epidemiological studies of the prevalence of asthma is to assess the risks associated with the various factors that evoke asthma. Therefore, questionnaires with high specificity and low sensitivity are more useful measures rather than with a lower specificity and high sensitivity. To the contrary, Smeeton et al. reported that the low coincidence between the standardized questionnaire and the postdemonstration questionnaire of asthma decreases the usefulness of this method for assessing the prevalence of asthma. The prevalence following the demonstration were 30–60 percent lower than those from the standardized questionnaire [29]. If we considered the prevalence of postdemonstration questionnaire as appropriate numbers of asthma, the prevalence of asthma reported by standardized questionnaires may be decrease. Of the questions, three items—attacks of wheezing, exercise-induced dyspnea, and allergen-induced dyspnea—were relatively well correlated with the presence of asthma. The high correlation with asthma symptoms suggests that those questions are closely related to the pathophysiology, which involves inflammation of pulmonary airways and bronchial hyper-responsiveness [30]. Our selective questionnaire had a relatively high negative predictive value (NPV) of over 82% despite a very low positive predictive value (PPV). This high NPV is a better asthma indicator for use in epidemiological studies. The items that differentiated asthmatics from non-asthmatics after multivariate logistic regression were exercise-induced dyspnea, recurrent attacks of wheezing, and pollution induced dyspnea (OR = 2.3, CI 1.5 to 3.5; OR = 2.0, CI 1.3 to 3.0; OR = 2.0, CI 1.3 to 3.0) respectively. On the contrary, questions about nocturnal cough or dyspnea and upper respiratory symptoms of more than 10 days’ duration were not able to discriminate between asthma and other respiratory conditions because these symptoms may be frequently followed by upper or lower respiratory infections and therefore have low predictability in terms of differentiating asthmatics from non-asthmatics. Shin et al. reported that a cutoff point of the total symptom score equal to or greater than the four questions was associated with the highest sensitivity (96%) and specificity (100%) [31]. However, their study involved fewer than 50 subjects, possibly introducing population bias. They also demonstrated that with an increased cutoff, the sensitivity decreased continuously, while the specificity remained ~100%. However, our study showed somewhat different results for a total score of ≥2, which had a sensitivity of 86.3% and a specificity of 20.4%. However, as the cutoff point increased, sensitivity decreased continuously from 98.4% to 18.5%, while specificity increased from 9.4% to 91.9%. In epidemiological surveys, a high specificity results in more effective detection of asthma and a high cutoff is more favorable for differentiation of asthmatics from non-asthmatics. Kim et al. reported the prevalence of childhood asthma based on questionnaires regarding asthmatic symptoms in Korea, and demonstrated that the sensitivity and specificity of wheezing, exercise induced dyspnea, and nocturnal dyspnea were 56.3%, 41.8%, and 37.9% vs. 69.0%, 41%, and 79%, respectively [32]. In the present study on adult asthma, the sensitivity and specificity of wheezing were similar to those in childhood asthma; however, the sensitivity of exercise-induced dyspnea in adult asthma was higher than that in childhood asthma, 41.8% vs. 70.2%, respectively. Therefore, exercise-induced symptoms may be more useful for diagnosis of adult than childhood asthma. In present study, exercise-induced dyspnea showed highest sensitivity (70.2%) and PPV (86.2%) among questions and this item is strongly recommended for diagnosing adult asthma. Zhong et al. reported that ~45% of asymptomatic students with a positive BHR developed asthma in the following 2 years [33]. In present study, a PC20 ≤ 50 mg/ml exhibited a higher sensitivity than a PC20 ≤ 25 mg/ml. The PC20 ≤ 50 mg/ml value is better at detecting mild asthma, particularly in cases of frequent or prolonged mild respiratory symptoms, such as chronic cough, which is frequently regarded as a symptom of simple upper respiratory infections in a clinical setting. The result of negative MBPT does not always exclude clinical asthma because the results of MBPT vary according to the purity of methacholine and the protocols. Therefore, in cases of patients with a negative MBPT and significant respiratory symptoms related to asthma, patients should be followed up and probably need to repeat MBPT at other times. On the other hand, subjects with a positive MBPT and no asthma symptoms must also be followed up because some subjects will likely be confirmed to be asthmatics within several years. Therefore, the asymptomatic subject with a positive BHR must be followed carefully to detect asthma early on. Early diagnosis of asthma may be very helpful to prevent asthma patients from progressing to permanent airway remodeling which can no longer be controlled by conventional asthma treatments. The questionnaire used in the present study may be suggested that it is a relatively convenient, accurate and cost-effective strategy for differentiating asthmatics from non-asthmatics. However, our study had several limitations. First, one major limitation is that there was no healthy control group. This problem probably make it somewhat difficult to argue that it is possible to calculate sensitivity and specificity of a symptom questionnaire to detect specific disease. Second, present study was performed at only one university hospital placed in a large city with relatively severe air pollution and a high density of population. Several environmental factors, such as economic state, the situation of air pollution, and the age of subjects, might have influenced our results. Third, this study included relatively small numbers of patients for an epidemiological survey. Despite its weaknesses, the major strength of this study is that elucidate the clinical validity of a selectively chosen questions recommended by GINA for diagnosing asthma in the general adult population. Especially, among five items, exercise-induced dyspnea, recurrent attacks of wheezing, and pollution induced dyspnea are more useful to differentiate asthmatics from non-asthmatics. Therefore, these three items may be adjusted to diagnose asthma more frequently than other questions.

## Conclusions

Present study showed that questionnaire which is properly matched with asthma like symptoms may be useful acceptable screening method to diagnosis asthma when MBPT is not available such as private clinics and epidemiological studies. A randomized large-scale study is needed to confirm our findings and the clinical usefulness of our methods in a private clinic or epidemiological survey.
